# Avoid overstepping the bounds of evidence: the role of the orthodontist in managing pediatric Obstructive Sleep Apnea

**DOI:** 10.3389/froh.2024.1486573

**Published:** 2024-11-06

**Authors:** Daniel J. Rinchuse, Andrea Boggio, Antonio Manni, Mauro Cozzani

**Affiliations:** ^1^ Advanced Education Program in Orthodontics and Dentofacial Orthopedics, Seton Hill University, Greensburg, PA, United States; ^2^ Department of Dentistry, Vita-Salute San Raffaele University, Milan, Italy; ^3^ Istituto Giuseppe Cozzani, La Spezia, Italy

**Keywords:** airway, OSA, orthodontics, evidence, breathing disorder

## Abstract

**Introduction:**

Pediatric Obstructive Sleep Apnea (OSA) is a common sleep-related breathing disorder often linked to distinct craniofacial features and malocclusions. While orthodontic treatments, particularly maxillary expansion and mandibular advancement, have been suggested for managing this condition, the results remain controversial and are based on low-quality evidence. This paper aims to summarize the ongoing debates on this topic by reviewing relevant literature and highlighting the role of the orthodontist in diagnosing and managing OSA in daily clinical practice.

**Discussion and conclusions:**

According to the present review, there is insufficient evidence to either confirm or deny the effectiveness of oral appliances for treating pediatric OSA due to significant methodological limitations, such as small sample sizes, inadequate control groups, short study durations, and a lack of long-term follow-up. Additionally, this condition cannot be diagnosed solely based on craniofacial morphology, but an interdisciplinary evaluation is strictly required. In addition, orthopedic treatment may be considered only as an adjunct therapy for children with craniofacial anomalies increasing the risk for OSA, and the combination of multiple therapeutic approaches may be necessary to achieve effective treatment outcomes.

## 1 Introduction

Obstructive Sleep Apnea (OSA) is a multifactorial breathing sleep disorder, consisting in partial or intermittent complete blockage of the upper airways during sleep, which affects about 1%–4% of children, with a slightly higher prevalence in males than in females (1). This causes a reduction or absence of airflow despite continuous respiratory effort and is usually associated with reduced peripheral oxygen saturation and hypercapnia (2). To date, several risk factors, such as adenotonsillar hypertrophy, obesity, neuromuscular tone, rostral fluid shifts, genetic diseases like Down syndrome, asthma and allergies have been identified. Moreover, since this condition has been associated with some craniofacial features (3–5), such as maxillary transverse deficiency, mandibular hypoplasia or retrusion, hyperdivergent skeletal pattern (6), some authors have promoted orthodontic treatment for the management of child breathing problems (7–9), however such results are quite controversial, and most studies are based on low quality of evidence. Therefore, the aim of this paper is to summarize the current debates on this topic by reviewing relevant literature and highlighting the role of the orthodontist in diagnosis and management of OSA in daily clinical practice.

## 2 OSA and orthodontics

In 2019 because of the controversial role that dentistry and particularly orthodontics played in the diagnosis, management, and treatment of OSA and airway problems, the American Association of Orthodontists taskforce published a white paper on OSA and orthodontics (10). The main point from this paper was, “Orthodontists should not assume responsibility for the definitive diagnosis of OSA.” Dental professionals can assume a screening role such as the use of the STOP-Bang questionnaire for adults and the Pediatric Sleep Questionnaire (PSQ) for children since the diagnosis of OSA in children is confirmed by the gold standard PSG (polysomnography). Therefore, the definitive diagnosis is appropriately made by a physician.

At least four relatively recent narrative reviews addressed the OSA debate (11–14). Rinchuse (12) challenged the “airway friendly” orthodontic movement from an evidence-based perspective and suggested that studies should explore the sources and reasons for misinformation being circulated to dental and orthodontic practitioners, and even patients. Kazmierski (14) emphasized the role of the orthodontist as screening for obvious OSA signs and symptoms and if necessary to make appropriate referral to a physician. To emphasize that the diagnosis and treatment of OSA is a medical condition, not dental, Kandasamy factiously added, “If our colleagues want to play medical doctor, then they should consider going to medical school. If our colleagues want to carry out orthodontic treatment to treat medical problems based on anecdotal claims and beliefs unsubstantiated by the evidence, such as expansion and growth modification at all costs, then this is not only unethical but grossly misleading to the public.”

The recent American Academy of Dental Sleep Medicine (AADSM) consensus panel of 12 experts reviewed literature relating to numerous therapies for OSA and snoring in both adults and children (15). This consensus paper is believed to be the first to evaluate novel therapies in both children and adults. Modalities of interventions reviewed were: myofunctional orthodontics, expansion for maxillary constriction, myofunctional therapy for tongue motor immaturity, lingual and buccal releases for tethered tissue, ablative and nonablative lasers for elongated or edematous soft palate and adjacent tissue, and extractions. There was agreement among panelists that none of the reviewed therapies were considered appropriate as first-line single therapies. They however may be appropriate as possible secondary, tertiary, or rescue options. Limitations of this paper were mentioned as well as the requirement for additional long-term RTCs with larger sample sizes.

The contretemps regarding OSA and airway parallels the early controversy concerning the diagnosis and treatment of TMDs (Temporomandibular Disorders), which centered on a dental, occlusal, gnathological, and jaw function philosophical perspective. Now the definition of TMD has involved into a more complex disorder as a group of muscular and neuromuscular conditions that include the muscles of mastication, the TMJ, and associated structures. From an evidence-based perspective TMDs are now viewed from a medical and biopsychosocial perspective (16). Likewise, OSA is more complicated and multifactorial in etiology. Adenotonsillar hypertrophy, neuromuscular tone, obesity, rostral fluid shifts, genetic predisposition influencing craniofacial anatomy and other related factors account for etiologies. In particular, adenotonsillar hypertrophy, which typically occurs during the period of significant lymphoid tissue growth, between the ages of 8 and 10, is recognized as the leading cause of sleep-disordered breathing (SDB) in children (17). Also obesity is considered an independent risk factor for the onset and progression of sleep-disordered breathing (17), while a reciprocal interaction between Pediatric OSA and asthma has been reported, whereby each disease impacts the severity of the other (18). In addition to risk factors, collapsibility of the upper airway is influenced further by OSA severity, which is heterogeneous among patients with the disorder. Pediatric OSA can be generally categorized into mild [AHI or respiratory disturbance index (RDI) between 1 and <5 events per hour], moderate (AHI between 5 and <10 events per hour), and severe (AHI ≥10 events per hour). This wide range of presentation leads to variations in management approach and differences in treatment response (10).

Even though impaired neuromuscular tone is a more valid assessment of OSA than airway volume (10), airway volume still seems to be the focus of some contentious groups and individuals for the management and treatment of OSA. For instance, maxillary expansion in the transverse dimension and advancement of the maxilla and/or mandible in the sagittal is recommended for increasing airway volume whereas orthodontic extractions are condemned for reducing airway volume and causing harm.

Recently there have been claims even for maxillary expansion in children as early as 3 years of age (19). This advocacy for early expansion has been dispelled by both Rinchuse (12) and Kandasamy (13). Rinchuse argued that caution should be advised, especially aggressive treatment for young children, without high quality investigations and particularly “long-term follow-up.” Therefore, when evaluating expansion, or craniofacial growth modiﬁcation claims for pre-school children relative to airway development and decrease in the size of enlarged adenoids and palatine tonsils, it is imperative to consider long-term maintenance and stability. Successful short-term outcomes may have relative statistical significance, but no clinical significance, and no long-term stability and pertinence. For instance, as Lyle Johnston argued it may be a “mortgage on growth” like ostensibly successful early Class II Phase I treatments (20). Meaning there is a temporary effect that is paid back later with no overall net gain. Or a “Soft Tissue Paradigm” (21) in which the stability of results is related primarily to soft tissue pressure and equilibrium effects, with relapse seen years later, like very young children outgrowing treatment. Finally, the American Academy of Dental Sleep Medicine (AADSM) consensus panel concluded that maxillary constriction is not a factor that contributes to pediatric OSA. “There was insufficient evidence to support RME as a treatment to cure pediatric OSA and stressed that expansion should only be considered in those patients who demonstrate maxillary constriction, independent of having pediatric OSA” (15). In addition, extractions were evaluated as to whether they decrease airway and tongue volume leading to pediatric OSA. The overall conclusion was that extractions in children are not a risk factor for OSA. Malocclusion and craniofacial morphologies identified as predisposing for OSA and airway problems include “retrognathia, long and narrow faces, dolichocephalic facial type, narrow and deep palate, steep mandibular plane angle, anterior open bite, midface deficiency, and lower hyoid position. It should be noted, however, that the strength of the relationship between these craniofacial morphologies and the development of OSA is not well established” (10).

Functional appliances have been advocated as useful in the treatment of breathing sleep disorders, however, while mandibular advancement has shown some benefits in treating primary snoring (22) and mild-to-moderate Obstructive Sleep Apnea (23) in adults, the situation appears more complex in pediatric patients. Currently, there is no robust, evidence-based support for its benefits in children, if compared with adenotonsillectomy (AT), which remains the first-line therapy for pediatric OSA (24). Notably, no studies have demonstrated that functional appliances are superior to AT in children with an Apnea-Hypopnea Index (AHI) >10 (15).

Furthermore, while the persistence of sleep-disordered breathing after surgery has been linked to pre-existing craniofacial characteristics that contribute to a reduced upper airway size (25, 26), a clear cause-and-effect relationship has not been firmly established (27). In fact, systematic reviews (28–32) indicate that due to strong limitations such as small sample sizes, inadequate control groups, short study durations, and a lack of long-term follow-up, there is insufficient evidence to either confirm or refute the effectiveness of oral appliances for treating pediatric OSA. Moreover, although some studies reported the reduction of at least 50% in respiratory events in compliant patients, this treatment alone does not guarantee a cure for OSA (AHI <1 event/h) and the conclusion was, “It may be necessary to combine therapies to achieve a cure” (28).

While most research has focused on mandibular advancement, few papers have evaluated the effect of maxillary protrusion. The conclusion of a systematic review and meta-analysis on this topic indicated that “maxillary protraction appliances can only increase pharyngeal airway dimensions in the short term” (33). Unfortunately, analyzing changes in posterior airway space through 2D or 3D radiography is insufficient for evaluating OSA, as it lacks pre- and post-intervention AHI data and does not accurately reflect the supine sleep airway anatomy due to altered physiological states and head positions (15). Accordingly, OSA cannot be confirmed by craniofacial morphology alone and, based on current scientific evidence, orthopedic treatment might be considered in specified cases as an auxiliary treatment for children with craniofacial anomalies that are risk factors for OSA, but combining multiple therapies might be necessary to achieve successful treatment outcomes.

Nevertheless, orthodontists should be well-versed in the signs and symptoms of Obstructive Sleep Apnea (OSA) and capable of conducting clinical risk assessments for the condition. It is highly recommended that orthodontists refer patients who may be at risk to a qualified physician for a definitive diagnosis (10). In cases where a physician identifies a skeletal discrepancy contributing to pediatric OSA, orthodontists may play a key role in treatment if the patient is referred back to them for intervention.

## 3 Discussion and conclusions

This paper has provided a concise, provocative review of OSA and airway problems particularly in children. Regrettably knowledge alone may have little impact on changing attitudes and certainly not behaviors. Biases and misconceptions are still pervasive in medicine and dentistry (34). Maybe even in the health sciences, it may be as Cavett Robert (35) pointed out in reference to social proof that, “95% of people are imitators and only 5 percent initiators, people are persuaded more by the actions of others than any proof we can offer.” Adherence to unproven claims can have serious health related consequences such as increased burden of care (time, finances, extended treatments), and inappropriate and unnecessary treatments. As dentistry and orthodontics move closer to being as an evidence-based health care profession, we may observe less ambiguity, confusion and uncertainty.

Based on the above considerations, the following conclusions can be drawn:

• The diagnosis of OSA in children is confirmed by the gold standard PSG (polysomnography) and by a physician.
• Dental professionals and orthodontists may have a supporting role to screen for OSA.
• Airway volume is not a reliable and valid assessment tool for the diagnosis of OSA.
• Maxillary expansion solely for OSA is inappropriate.
• Extractions in children are not a risk factor for OSA.
• Malocclusion and craniofacial morphologies may be associated with OSA and airway problems, but not a cause and effect.
• An interdisciplinary approach to treating Obstructive Sleep Apnea (OSA) ensures that patients receive the most comprehensive and effective care.
